# Fourier Channel Attention Powered Lightweight Network for Image Segmentation

**DOI:** 10.1109/JTEHM.2023.3262841

**Published:** 2023-03-29

**Authors:** Fu Zou, Yuanhua Liu, Zelyu Chen, Karl Zhanghao, Dayong Jin

**Affiliations:** UTS-SUStech Joint Research Centre for Biomedical Materials and DevicesDepartment of Biomedical EngineeringSouthern University of Science and Technology Shenzhen Guangdong 518055 China; Faculty of ScienceInstitute for Biomedical Materials and Devices (IBMD), University of Technology Sydney 1994 Ultimo NSW 2007 Australia

**Keywords:** Medical image segmentation, Fourier channel attention, residual unit, pathological section, Clinical and Translational Impact Statement- Medical image segmentation can be used to measure the position and size of human tissues or lesions making the changes of anatomical or pathological structures in the image clearer It plays a vital role in computer-aided diagnosis and intelligent medical treatment At the same time quantitative measurement and analysis of relevant imaging indicators before and after treatment will help doctors diagnose follow up or revise the treatment plan for patients

## Abstract

The accuracy of image segmentation is critical for quantitative analysis. We report a lightweight network FRUNet based on the U-Net, which combines the advantages of Fourier channel attention (FCA Block) and Residual unit to improve the accuracy. FCA Block automatically assigns the weight of the learned frequency information to the spatial domain, paying more attention to the precise high-frequency information of diverse biomedical images. While FCA is widely used in image super-resolution with residual network backbones, its role in semantic segmentation is less explored. Here we study the combination of FCA and U-Net, the skip connection of which can fuse the encoder information with the decoder. Extensive experimental results of FRUNet on three public datasets show that the method outperforms other advanced medical image segmentation methods in terms of using fewer network parameters and improved accuracy. It excels in pathological Section segmentation of nuclei and glands.

## I. Introduction

Medical image processing becomes essential in computer-aided diagnosis(CAD) [1], image-guided surgery(IGS) [2], and tumor radiation therapy [3]. With increased resolutions that modern biomedical imaging instruments can offer, segmentation algorithms have been developed and widely applied in various imaging modalities, including X-ray [4], Computed Tomography (CT) [5], Magnetic Resonance Imaging (MRI) [6], [7], endoscopy [8], wireless capsule endoscopy [9] and high-throughput imaging techniques, like histopathology and electron microscopy (EM) [10]. In microscopic applications, cellular imaging and subcellular region segmentation are essential to characterize cellular dynamics under normal and pathological conditions [11], study drug discovery, and evaluate the efficacy of drug treatments [12].

Conventional methods, based on morphological operations to segment cells or intracellular compartments, lack sufficient accuracy in segmentation results and are inefficient in generalizing the new datasets. Deep-learning-based segmentation has been rapidly developed with improved accuracy and can be adapted to a variety of pathological slice cell maps. Al-Kofahi et al. use a cascade of multiple layers of nonlinear processing units for feature extraction and transformation, forming hierarchical features from low-level to high-level to perform cell segmentation [13]. Greenwald et al. constructed TissueNet containing more than 1 million manually labeled cells to greatly improve the accuracy of the cell segmentation network [14]. Estibaliz et al. embedded the cell segmentation function into ImageJ so that experts without the machine learning foundation can easily use this function for scientific research [15]. These works have promoted the development and application of cell segmentation algorithms.

### A. Related Work

Methods based on convolutional neural networks (CNNs) have overcome many shortcomings of traditional algorithms in the field of medical image segmentation. Compared with traditional methods, deep learning has higher accuracy and robustness, even under dynamic backgrounds, resolution, or light source conditions. Deep learning has good flexibility, as CNN models and frameworks can train multiple types of datasets and are more versatile. Deep learning is end-to-end learning, and the network can automatically find the most descriptive and obvious features without relying on the judgment of engineers and long-term debugging and error handling [16].

Long et al. [17] proposed a Fully Convolutional Network (FCN) that further extends from image-level classification to pixel-level classification for semantic segmentation. FCN achieves end-to-end segmentation, but the segmentation accuracy is not enough. Based on a classic encoder-decoder idea, U-Net has been developed from FCN by Ronneberger et al. [18]. Compared with FCN, U-Net has a symmetrical structure and skip connections. The downsampling of U-Net increases the receptive field and upsampling increases the resolution of high-level abstract features and fuses with low-level surface features through skip connections. This allows features of different scales to be fused, enabling multi-scale prediction and deep supervision.

As the network deepens, vanishing/exploding gradients and network degradation problems are prone to occur. ResNet [19] is a milestone in the history of CNN image processing, winning first place in classification and object detection in the 2015 ImageNet competition. Jha et al. proposed that ResUNet++ performed well on polyp segmentation which demonstrated a 10% improvement compared to the widely used UNet baseline on the Kvasir-SEG dataset [20].

The attention mechanism is to imitate the process of paying attention to the Region of Interest. We can adaptively make the network focus on more effective features by exploiting the interdependencies between channel or spatial domain features. This enables the network to extract richer information in limited steps. At the same time, it improves the interpretability of the network and has the potential to increase clinician acceptance and trust in predictions given by AI algorithms [21]. The core idea of the attention module is to adaptively acquire the importance of each feature through learning, automatically enhance useful features and suppress unimportant features. Gu et al. make extensive use of multiple attention in the CNN architecture, synthesizing spatial attention, channel attention, and scale attention to be aware of the most important spatial locations, channels, and scales for more accurate medical image segmentation [21]. The SE block allows the network to perform feature recalibration by explicitly modeling the interdependencies between channels of convolutional features [22] Yu et al. specifically designed a smooth network using Channel Attention Block and Global Average Pooling to choose more discriminative features for semantic segmentation [23].

Frequency domain conversion is very common in traditional image processing because the high and low frequency of the image represents the contour and detail information of the image, which is very important for image processing. Yu et al. proposed importing spatial frequency domain instead of RGB image into CNN to extract features. This method can significantly reduce the amount of data transmission and improve the accuracy of the model to a certain extent [24]. They successfully applied frequency domain information to CNN. Qin et al. [25] proposed FcaNet with a multi-spectral attention module, which popularizes the existing channel attention mechanism in the frequency domain, uses DCT to put the characteristics of different frequencies into different channels, and then uses the attention mechanism to multiply the weight of different channels by the corresponding channels in the spatial domain. We refer to the Fourier channel attention (FCA Block) of Qiao et al. [26], and increase the value of the high-frequency part after Fourier transforms enhancing the contributions of high-frequency components. While FCA is widely used in image super-resolution with residual network backbones, its role in semantic segmentation is less explores. Here we study the combination of FCA and U-Net to segment biomedical images.

### B. Our Approach

Inspired by these successful approaches above, we draw on the encoder-decoder structure and use the Residual Unit as the basic structure. The encoder downsamples three times, reducing the size of the feature map by half each time, and the decoder symmetrically upsamples three times symmetrically, enlarging the feature map by half each time. When down-sampling, the encoder will obtain a high-level semantic feature map as the depth deepens. Up-sampling combines the low-level semantic features of the encoder with the corresponding layer to enrich the overall features while restoring the resolution. Every Residual Unit is composed of two layers of FCA Block and the BN layer that follows it. FCA Block gives the frequency domain information after Fourier transforms as the weight of the channel to the spatial domain through the attention mechanism so that the spatial domain can obtain richer information to improve the segmentation performance. We have evaluated our method on three publicly available datasets. Our experiments show that our model outperforms most of the baseline models and achieves comparable performances with SOTA nucleus and gland segmentation, and the network significantly reduces the number of parameters and computational costs while ensuring segmentation performance.

In summary, the contributions of the paper include:

**The combination of FCA and U-Net –** We propose a novel FRUNet architecture that leverages the advantages of FCA blocks and residual blocks based on an excellent U-Net network. These ingenious structures enable the network to quickly extract useful and effective features, accelerate network convergence, and improve training efficiency while improving segmentation accuracy.

**Systematic evaluation –** The effectiveness of FRUNet has been evaluated using three public datasets: Kaggle 2018 Data Science Bowl, GlaS, and MoNuSeg. These three datasets are mainly used for nucleus and gland segmentation, and each of them has its characteristics to verify the network’s robustness in handling diverse nuclei, its ability to segment irregular and dense nuclei, and its capability to handle small sample datasets.

**Network lightweight —** The FRUNet parameters is several times smaller than that of the current segmentation networks, which results in near SOTA performance with far fewer computational cost. Because the network has a strong feature extraction ability and fast convergence, it can obtain sufficient accuracy with a small number of layers and channels.

## II. Method

### A. Architecture of FRUNet

**FIGURE 1.**
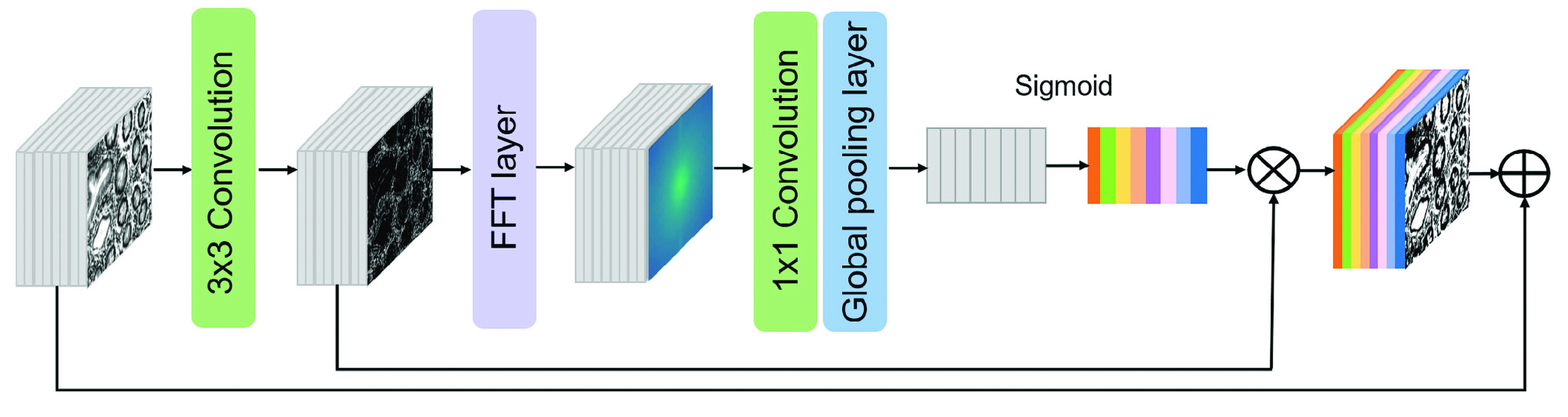
The architecture of FCA Block.

The overall design is illustrated in Fig. 1. We utilize features in the frequency domain with channel attention. The frequency-domain information represents the change of the gray values relative to the adjacent points in the image. On the edge, the intensity of the image changes drastically, as the high-frequency information of the image. In traditional image processing, operations such as frequency domain filtering are often used to process images, while little attention has been paid to the Fourier domain so far in deep learning. Chang et al. speculate that using the frequency content difference between different features in the Fourier domain may enable the super-resolution reconstruction precisely and efficiently because it can learn high-frequency hierarchical information more effectively [26]. As high-frequency information is also important for image segmentation, we apply an FFT layer to extract features in the frequency domain.

The squeeze-and-excitation (SE) block improves model accuracy by weighting different channels to emphasize the important features. To make the network pay attention to the high-density information of the features, we referenced the SE block and proposed a Fourier channel attention block. The extraction of frequency domain information and the channel attention mechanism greatly improve the richness of information [22]. We combine frequency domain features with the advantages of SE-Net to make the network more sensitive to the important frequency domain features.

Therefore, we designed the FCA block to focus on the high-frequency information of the edge. The FCA Block structure first passes the input feature map through two layers of $3\times 3$ convolution, and each layer of convolution is followed by a Gelu activation layer to extract spatial domain information, and then Fourier transform is performed, $1\times 1$ convolution to extract frequency domain information and use ReLU as the activation function. The feature map is then compressed by global average pooling to obtain a global understanding of each channel. After the global average pooling of frequency domain information, the weight of each channel is obtained through the Sigmoid activation function. Layers that contribute more to the network have greater weight values and then the frequency domain feature weight is adaptively assigned to spatial domain feature maps by multiplying the weights with the spatial feature map correspondingly. Finally, the feature map with frequency domain weight is added to the input feature map of this block to further fuse more feature information.

**FIGURE 2.**
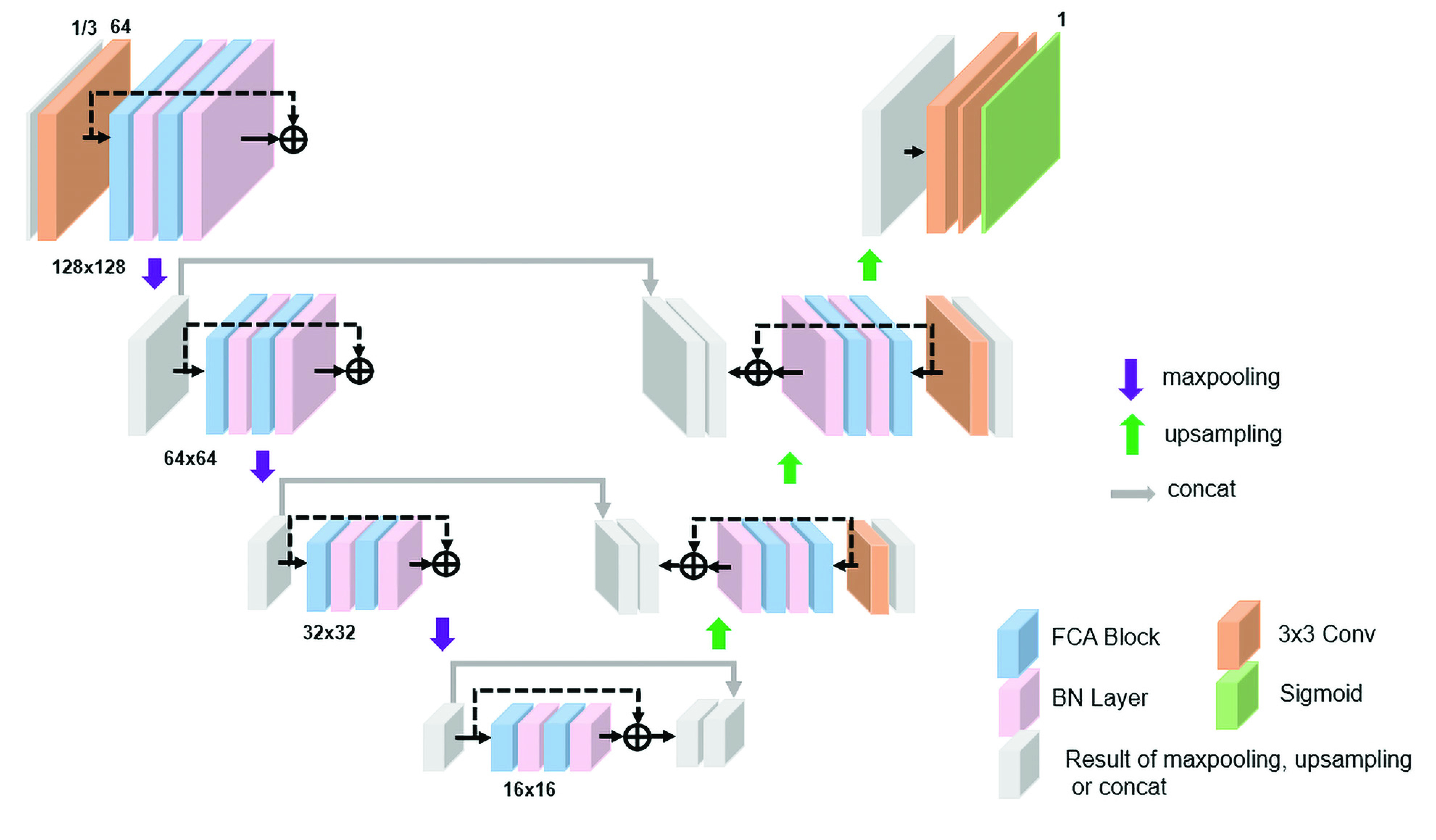
Block diagram of the proposed FRUNet architecture. The network is mainly composed of FCA Block, Batch normalization, and convolution layers. The thickness of the small square represents the number of channels. Except for the input layer and output layer, the number of other layers is 64. Each max pooling shrinks the feature map in half and each upsampling doubles.

The block diagram of FRUNet is shown in Fig. 2, which is mainly composed of FCA blocks and residual blocks to form a U-shaped network. The FCA block can extract both spatial and frequency domain features in a small module. The attention module allows the network to automatically learn useful features to greatly improve the efficiency of feature extraction. Residual block enables the network to be deep and obtain more high-level useful information. Because skip connections help information spread without degradation, the residual network converges faster with the same number of layers, and a deeper network can be used. These advantages suggest the ResNet structure is the basic module of FRUNet. The residual block consists of two FCA Blocks where each block is followed by the Batch Normalization layer. The input feature map is added to the feature map that has passed through these four layers, which helps the spread of information and can greatly reduce parameters to optimize the network design, making the model easier to train and improving the accuracy of segmentation.

Input channels are determined as 3 channels or 1 channel depending on whether the input image is an RGB or grayscale image. The $3\times 3$ convolution was used for feature extraction and channel size adjustment shown as the orange rectangular block. The last layer of the network uses $1\times 1$ convolution and Sigmoid activation functions to get the segmentation map. As shown in the figure that with the proceeds of downsampling, the size of the feature map gradually decreases from $128\times 128$ to $16\times 16$, but the number of channels remains 64. We try to increase the channels of feature maps as the layers deepen, but the segmentation results do not change much. The experiment shows that 64 channels are enough to extract useful features. Adding channels here only increases the number of network parameters, and the effect is not significantly improved, as shown in TABLE 5.

**TABLE 1:** Medical image segmentation datasets used in our experiments.

Dataset	Images	Input size	Train	Valid	Test
2018 Data Science Bowl [27]	670	$256\times 256$	400	170	100
GlaS [28]	165	$128\times 128$	68	17	80
MoNuSeg [29,30]	51	$512\times 512$	30	7	14

**TABLE 2:** Results on the 2018 data science bowl.

Method	DICE	mIoU	Recall	Precision	Parameters
U-Net [18]	0.9080	0.8314	0.9029	0.9130	7.11M
U-Net++ [32]	0.7705	0.5265	0.7159	0.6657	9.04M
ResUNet++ [20]	0.9098	0.8370	0.9169	0.9057	4.07M
Deeplabv3+ [33]	0.8857	0.8367	0.9141	0.9081	2.14M
DoubleUNet [34]	0.9109	0.8429	0.9278	0.9020	29.29M
ColonSegNet [35]	0.9197	0.8466	0.9153	0.9312	5.01M
MSRF-Net [36]	0.9224	0.8534	0.9402	0.9022	18.38M
FRUNet (Ours)	0.9253	0.8657	0.9441	0.9120	1.78M
FRUNet (5-fold)	0.9324	0.8764	0.9467	0.9212	1.78M

**TABLE 3:** Results on glas.

Method	F1 score	mIoU	Parameters
FCN [17]	0.6661	0.5084	12.5M
U-Net [18]	0.7778	0.6534	3.13M
U-Net++ [32]	0.7803	0.6555	5.32M
MedT [37]	0.8102	0.6961	1.4M
FRUNet (Ours)	0.8600	0.7454	1.78M
FRUNet (5-fold)	0.9153	0.8496	1.78M

**TABLE 4:** Results on MoNuSeg.

Method	DICE	mIoU	F1	Parameters
FCN [17]	–	0.2871	0.2884	12.5M
U-Net [18]	0.7707	0.6356	0.7795	31M
U-Net++ [32]	0.7616	0.6216	0.7737	36.17M
ResUNet [38]	0.7737	0.6351	0.7814	6.27M
FRUNet (Ours)	0.7894	0.6552	0.7967	1.78M
FRUNet (5-fold)	0.8099	0.6872	0.8099	1.78M

**TABLE 5:** Parameter experiment on the 2018 data science bowl.

The change in the network	DICE	mIoU	Precision	Recall	F1	Parameters
Channels increase with the layers(channel = 64,128,256)	0.9059	0.8356	0.9636	0.8602	0.9090	11.56M
4-layers(channel = 64)	0.9128	0.8458	0.9126	0.9221	0.9174	2.45M
Channel increased and 4-layers(channel = 64,128,256,512)	0.9219	0.8601	0.9276	0.9225	0.9251	45.61M
FRUNet(Ours)	0.9253	0.8657	0.9441	0.9120	0.9278	1.78M

### B. Loss Function and Evaluation Metrics

The segmentation of cells and gland is performed on the pixel level, and the samples are usually unbalanced. Therefore, two different loss functions are chosen in this work. The first is the cross-entropy loss function, which is commonly used to describe the difference between two probability distributions. However, when the number of foreground pixels is much smaller than the number of background pixels in the segmentation task, the model with only cross-entropy loss will be heavily biased towards the background, resulting in poor results. We further combine the Dice coefficient to calculate the similarity between two images, which is more suitable for unbalanced samples. The final loss function is described in Eq. (3) as follows:

\begin{align*} L_{BCE}&=\left ({y-1 }\right)\log \left ({1-\hat {y} }\right)-y\log \hat {y} \tag{1}\\ L_{DCS}&=1-\frac {2y\hat {y}+1}{y+\hat {y}+1} \tag{2}\\ Loss&=L_{BCE}+L_{DCS} \tag{3}\end{align*}

where y is the ground truth value and $y\hat{}$;s the predicted value. The sum of these two loss functions is used to minimize the gradient between the prediction map and the annotations.

The following metrics are used for the quantitative evaluation of our method with other recent methods, which are commonly used metrics in medical image segmentation.

\begin{align*} DICE&=\frac {2TP}{2TP+FP+FN} \tag{4}\\ mIoU&=\frac {1}{k+1}\sum \limits _{i=0}^{k} \frac {TP}{TP+FN+FP} \tag{5}\\ Recall&=\frac {TP}{TP+FN} \tag{6}\\ Precision&=\frac {TP}{TP+FP} \tag{7}\\ F1&=\frac {2\times Precision\times Recall}{Precision+Recall} \tag{8}\end{align*}

At the same time, we provide visual sample predictions of qualitative results to analyze why the proposed method performs better than other approaches.

### C. Dataset

We have used three medical image segmentation datasets to validate the performance of our proposed FRUNet network: Kaggle 2018 Data Science Bowl, GlaS, and MoNuSeg, which are publicly available from their official websites. The 2018 Data Science Bowl contained a large number of segmental nuclear images of different cell types obtained under a variety of conditions and varied in cell type, magnification, and imaging modality [27]. This verifies the robustness of the network to the segmentation of cells with large differences. GlaS consisted of 165 histological sections from 16-stage T3 or T42 colorectal adenocarcinoma stained with hematoxylin and eosin [28]. The dataset consists of tissue images of tumors of different organs from different patients, created by downloading H&E stained tissue images at $40\times $ magnification from the TCGA archive, all carefully annotated by experts. This dataset was used to validate the performance of the network for gland segmentation. MoNuSeg was obtained by carefully annotating tissue images of several patients with tumors of different organs and who were diagnosed at multiple hospitals [29], [30]. Given the diversity and high density of nuclear appearances across multiple organs and patients, the robustness of the network and the segmentation ability of high-density nuclei can be well validated. All of these datasets contain images and the corresponding GroundTruth annotated by expert pathologists. Due to the limited number of training images, we performed data augmentation, including rotation, zoom in or out, shifting, horizontal flip, and reflection. The detail of the datasets and their distribution during the experiment can be found in TABLE 1.

### D. Implementation Detail

Our experiment uses Keras neural network API and runs on TensorFlow [31] backend. In the experiment, we used some uniform hyperparameters. We set the batch size to 2, used the Adam optimizer to train the network, and set the learning rate to 0.0001. If saturation is reached in a few epochs during the training, we set the learning rate to 0.00005. All experiments were conducted on NVIDIA-RTX 3090 GPU.

## III. Result

In this section, we discuss the results of experiments conducted by our proposed network FRUNet. All 5-fold cross validation results are also in the corresponding results table.

### A. Comparison of the Network Performance

Kaggle 2018 Data Science Bowl dataset contains 670 images with image sizes $256\times 256$. in which 570 images were for training and 100 for testing. The images under various conditions are converted into grayscale images and then taken as the input of the network in their original size. The result of the proposed FRUNet and the comparison of the present result shows in TABLE 2. The evaluation results show that our proposed network achieves a DICE of 0.9253, mIoU of 0.8657, recall of 0.9441, and precision of 0.912 which outperforms the previous best-performing MSRF-Net in all matrics. We can observe that the predicted results are very robust, and has high segmentation accuracies for nuclei of different types, sizes, and densities.

**FIGURE 3.**
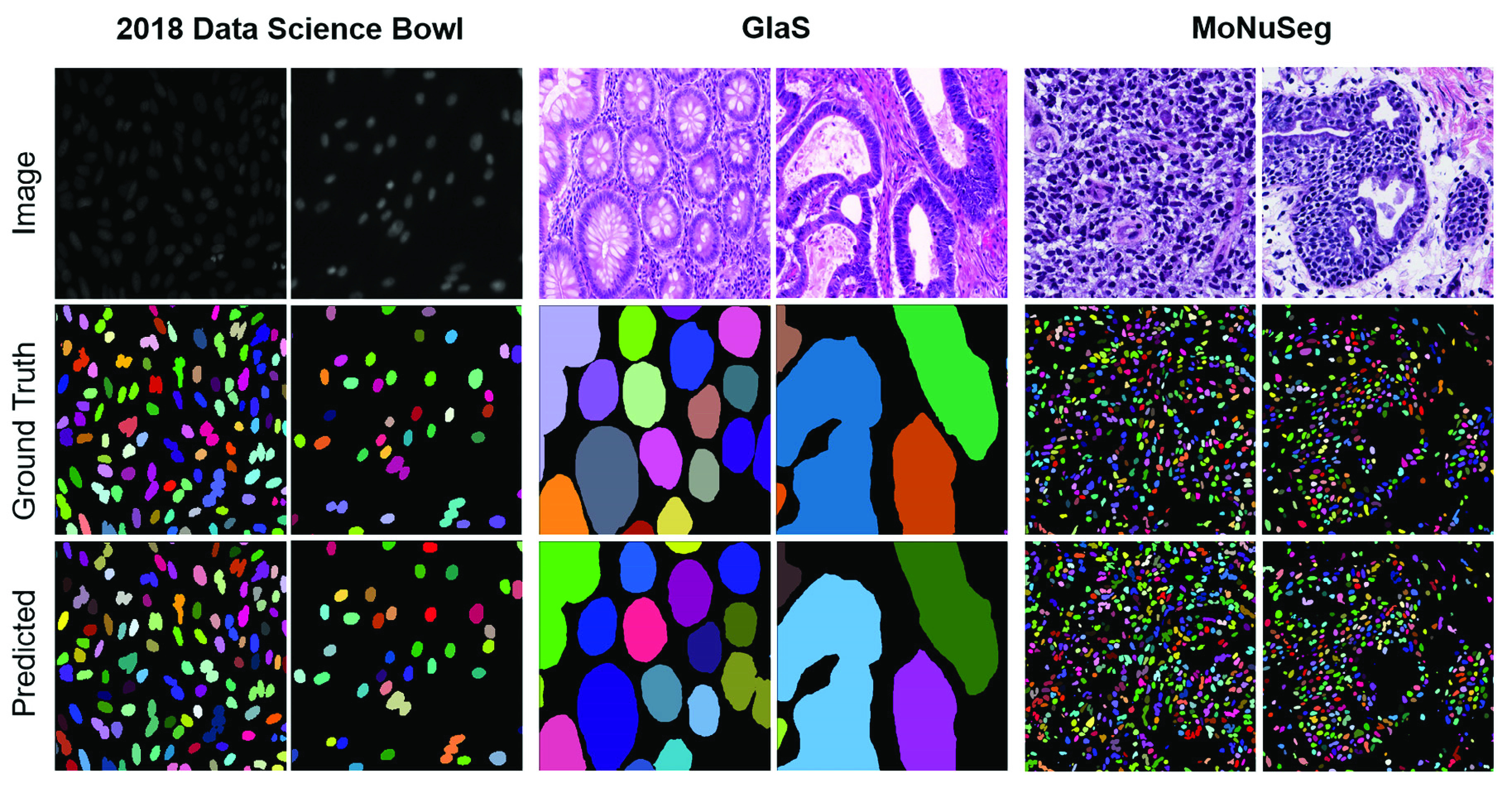
Qualitative results of FRUNet on sample test images from Kaggle 2018 data science bowl, GlaS, and MoNuSeg datasets. Two images are selected for each dataset to compare the prediction results with the ground truth.

GlaS contains 165 images with image size $775\times 522$, in which 85 images are used for training and 80 images are used for validation. The images are resized to $128\times 128$, which is same with the SOTA network. Since the RGB color has a certain auxiliary role for gland segmentation, the RGB images are used as the network input. The quantitative results for the GlaS are shown in TABLE 3. We can observe that our method achieved an F1 of 0.8600 and mIoU of 0.7454 which outperforms the SOTA networks. Although the glands are usually irregular in shape and similar in color to the background, our method achieves almost consistent results with the ground truth (Fig. 3). The result highlights the superior segmentation ability of the network. Our network achieves an improvement of nearly 5% on F1 score and 4.93% on mIoU compared to the current SOTA.

MoNuSeg contains 51 images with a size of $512\times512$, of which 37 images are for training and 14 for testing. The original images are converted into a grayscale image as input. From the quantitation result shown in TABLE 4, the proposed method achieves 0.7865 in DICE, 0.6499 in mIoU, and 0.7943 in F1 score which outperforms the backbone architectures. The dataset obtained from several organs with annotations of tens of thousands of individual nuclei validates the network’s ability to segment highly dense cells (Fig. 3). The results also demonstrate that our network can have good segmentation performance for small and complex datasets.

### B. Comparison of the Network Structure

From the result table, we can see that the number of network parameters of FRUNet is less than all networks except MedT. We conduct a series of comparative experiments on the dataset Kaggle 2018 Data Science Bowl. Based on FRUNet, change the number of channels, and the number of layers of the network to determine the optimal network structure. As shown in TABLE 5, the number of channels is always 64, and the number of layers is 3 layers is the combination with the best overall performance and the smallest network parameters. The size of the feature map of the U-Net network decreases as the network deepens, and channels are increased to retain more information. We imitated the characteristics of the U-Net and changed the number of channels of the 3-layer network to 64, 128, and 256. Although Precision is the best result, other parameters are much lower than other models, which means that when the number of channels is 64, enough features can be obtained for segmentation, and increasing the number of channels cannot improve the results or even have a negative effect. We have tried to increase the number of layers by changing the original 3-layer U-shaped structure to 4 layers, with the number of channels being 64. The segmentation performance is reduced compared to the best results. When the number of layers and channels are increased at the same time, the results are very similar to FRUNet, ignoring the influence of experimental errors, the results can be considered consistent, but the network parameters are increased by 25 times. All the above experiments show that the FRUNet is very ingenious, the structure is small and compact, and the information in the Fourier domain and channel attention can quickly obtain useful information, so the network parameters are greatly reduced.

### C. Ablation Study

**FIGURE 4.**
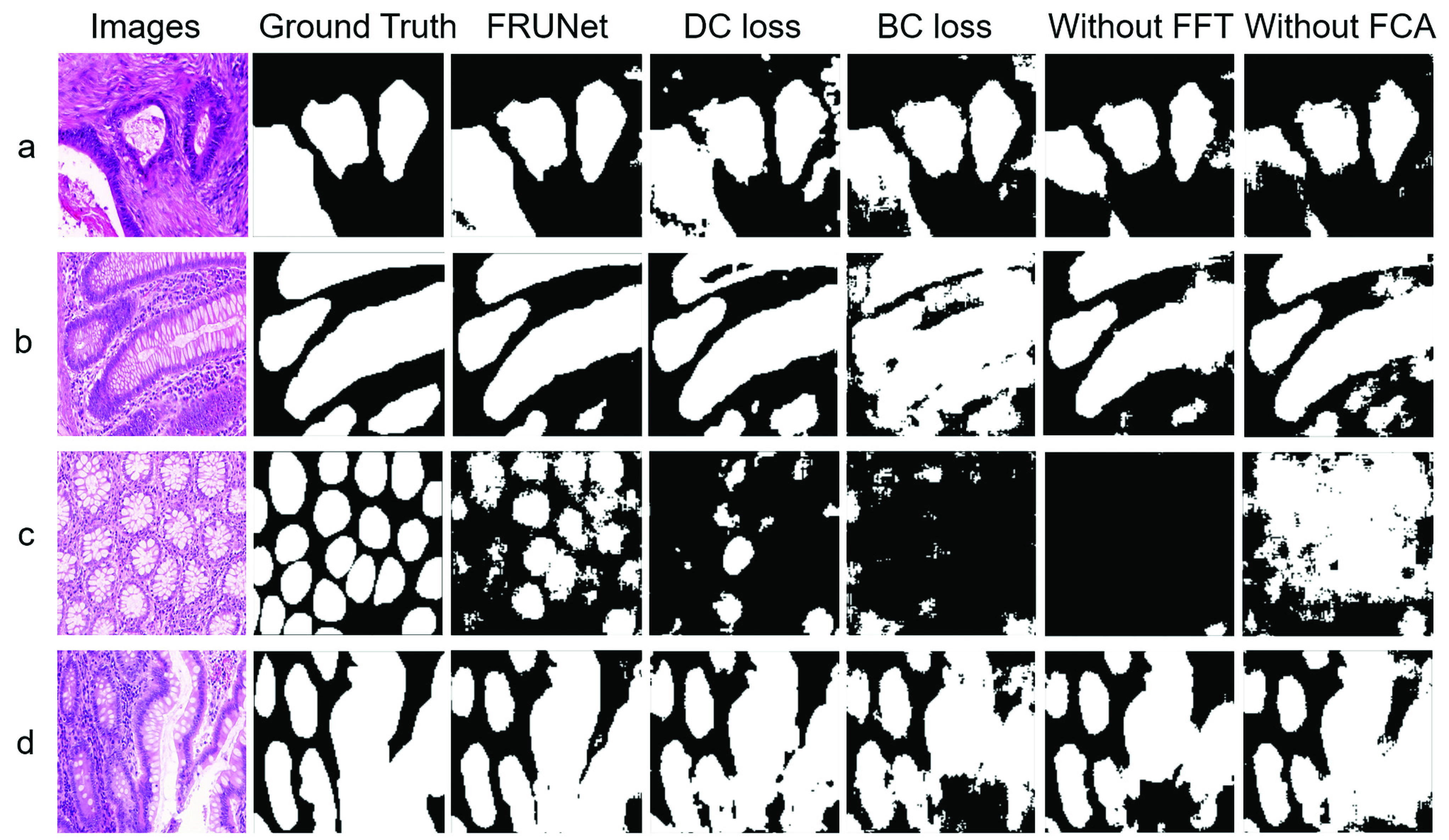
Qualitative result of ablation study of FRUNet on GlaS dataset. Select 4 samples a, b, c, and d in the GlaS dataset as the demonstration of the ablation experiment results.

We conduct extensive ablation experiments to verify the effect of each block of the network in the proposed method. In this experiment, the dataset we have used is GlaS. We have analyzed the effect of the Fourier transforms, the channel attention mechanism, and the different loss functions on the whole network separately. TABLE 6 shows the quantitative results of the ablation study, missing parts of the network make the difference in results noticeable. Firstly, we removed the Fourier transform of the FCA Block which equivalently led to this part becoming spatial domain channel attention, and that has resulted in the worst results on all the metrics except precision. It can be seen from the visualization results (Fig. 4) that the network with Fourier transform can better segment the glands with similar color or brightness to the background, as shown in sample c, almost all results are predicted to be false without the Fourier transform. We disabled the Fourier channel attention block to get a better result than in the first experiment. But relative to our proposed network, it will have more false positives. We can conclude from these two experiments that the spatial attention mechanism seems to have a negative effect on the network, but the attention mechanism in the Fourier domain has a positive effect, Samples b and d clearly show this feature. We use a combination of dice losses and binary cross-entropy losses for monitoring our proposed network during training. We first set the loss as Binary cross-entropy and followed by setting the loss of the Dice coefficient. Neither of them gives better results than the combination of the two.

**TABLE 6:** Ablation study on glas.

Method	DICE	mIoU	Recall	Precision	F1
FRUNet(Ours)	0.8469	0.7454	0.8505	0.8698	0.8600
Without Fourier transform	0.7198	0.6071	0.7248	0.8623	0.7876
Without FCA Block	0.7918	0.6735	0.7695	0.8597	0.8121
FRUNet with Loss = Binary cross-entropy	0.7445	0.6215	0.7579	0.8200	0.7900
FRUNet with Loss = Dice coefficient	0.8197	0.7197	0.8251	0.8723	0.8481

## IV. Discussion and Conclusion

**FIGURE 5.**
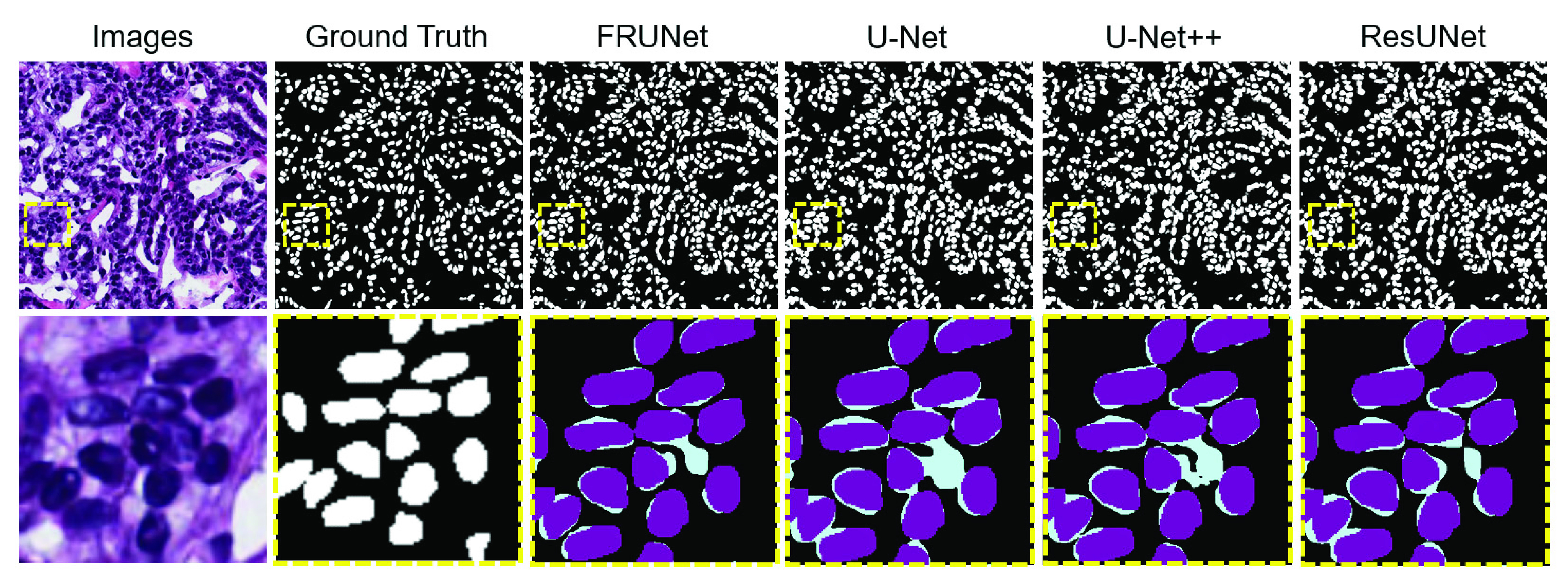
Qualitative results comparison on the MoNuSeg dataset. From the left: (1) Images, (2) Ground truth, (3) FRUNet, (4) U-Net, (5) UNet++., and (6) ResUNet, From the experimental results, we can say that FRUNet produces better segmentation masks than other competitors.

In order to help clinicians make an accurate diagnosis, it is necessary to segment some key objects in medical images and extract features from the segmented regions. This paper segmented three different tissues or cells from different patients and hospitals and verified the good segmentation performance and robustness of the proposed network, which helps doctors understand the pathological structure more clearly. We propose a lightweight network FRUNet for cell and gland segmentation that takes advantage of Fourier domain channel attention and encoder-decoder architecture. Such an ingenious structure allows the network to extract more useful features with a much smaller model size, resulting in high segmentation accuracy. FRUNet is based on the U-Net, FCA Block, and Residual block. FCA Block utilizes frequency-domain channel attention to give weights to the network’s spatial-domain features, making the network more sensitive to important features. Residual networks allow deeper neural networks that can address each encoder degradation problem, improve channel interdependencies, and save computational resources. The encoder-decoder structure of U-Net, combined with skip-connection, can make the high-level feature map integrate more low-level features, which is conducive to the accurate classification of pixels. FRUNet performs significantly on three datasets of cell and gland segmentation. We did experiments to compare our network with the current classical networks with better segmentation effects such as U-Net, U-Net++, and ResUNet, as shown in Fig. 5. Our proposed network can segment the edges more finely and can separate each nucleus individually, the segmentation accuracy is greatly improved. And the parameters are much less than other networks, which saves computational overhead and reduces training time to just a few hours. The model file of this network has been open source, and the website is https://zenodo.org/record/6919253#.Y2utT3ZByUk.

The ablation experiment demonstrates that the frequency domain information after the Fourier transforms and channel attention mechanism is added to greatly increase the segmentation accuracy. Dice coefficient loss can get better results, but combined with Binary cross-entropy loss, the best results of the experiment are obtained.

Our preprocessing of images is resizing, which may lose image information to a certain extent. The datasets we have chosen are the segmentation of nuclei and glands, and their segmentation results can outperform the performance of the SOTA network, but for other datasets, we can only achieve comparable performances with SOTA and cannot surpass the best.

## References

[1] M. Li, K. Zhao, C. Peng, P. Hobson, T. Jennings, and B. C. Lovell, “Deep adaptive few example learning for microscopy image cell counting,” in Proc. Digit. Image Comput., Techn. Appl. (DICTA), Nov. 2021, pp. 1–7.

[2] F. Alam, S. U. Rahman, S. Ullah, and K. Gulati, “Medical image registration in image guided surgery: Issues, challenges and research opportunities,” Biocybernetics Biomed. Eng., vol. 38, no. 1, pp. 71–89, 2018.

[3] D. A. Hormuth, A. M. Jarrett, and T. E. Yankeelov, “Forecasting tumor and vasculature response dynamics to radiation therapy via image based mathematical modeling,” Radiat. Oncol., vol. 15, no. 1, pp. 1–14, Dec. 2020. 10.1186/s13014-019-1446-2 PMC6941255 31898514

[4] E. El-Din Hemdan, M. A. Shouman, and M. Esmail Karar, “COVIDX-Net: A framework of deep learning classifiers to diagnose COVID-19 in X-ray images,” 2020, arXiv:2003.11055.

[5] T. Würfl, F. C. Ghesu, V. Christlein, and A. Maier, “Deep learning computed tomography,” in Proc. Int. Conf. Med. Image Comput. Comput.-Assist. Intervent. Cham, Switzerland: Springer, 2016, pp. 432–440.

[6] D. Chen, Z. Wang, D. Guo, V. Orekhov, and X. Qu, “Review and prospect: Deep learning in nuclear magnetic resonance spectroscopy,” Chem. Eur. J., vol. 26, pp. 10391–10401, Jan. 2020. 32251549 10.1002/chem.202000246

[7] A. S. Lundervold and A. Lundervold, “An overview of deep learning in medical imaging focusing on MRI,” Zeitschrift Medizinische Physik, vol. 29, no. 2, pp. 102–127, 2019. 10.1016/j.zemedi.2018.11.002 30553609

[8] W. Du, “Review on the applications of deep learning in the analysis of gastrointestinal endoscopy images,” IEEE Access, vol. 7, pp. 142053–142069, 2019.

[9] S. Soffer, “Deep learning for wireless capsule endoscopy: A systematic review and meta-analysis,” Gastrointestinal Endoscopy, vol. 92, no. 4, pp. 831–839, Oct. 2020. 32334015 10.1016/j.gie.2020.04.039

[10] J. P. Horwath, D. N. Zakharov, R. Mégret, and E. A. Stach, “Understanding important features of deep learning models for segmentation of high-resolution transmission electron microscopy images,” npj Comput. Mater., vol. 6, no. 1, pp. 1–9, Jul. 2020.

[11] C. L. Chaffer and R. A. Weinberg, “A perspective on cancer cell metastasis,” Science, vol. 331, no. 6024, pp. 1559–1564, Mar. 2011. 21436443 10.1126/science.1203543

[12] X. B. Zhou and S. T. C. Wong, “High content cellular imaging for drug development,” IEEE Signal Process. Mag., vol. 23, no. 2, pp. 170–174, Mar. 2006, doi: 10.1109/MSP.2006.1598095.

[13] Y. Al-Kofahi, A. Zaltsman, R. Graves, W. Marshall, and M. Rusu, “A deep learning-based algorithm for 2-D cell segmentation in microscopy images,” BMC Bioinf., vol. 19, no. 1, Oct. 2018, doi: 10.1186/s12859-018-2375-z. PMC6171227 30285608

[14] N. F. Greenwald, “Whole-cell segmentation of tissue images with human-level performance using large-scale data annotation and deep learning,” Nature Biotechnol., vol. 40, no. 4, pp. 555–565, Apr. 2022, doi: 10.1038/s41587-021-01094-0. 34795433 PMC9010346

[15] E. Gómez-de-Mariscal, C. García-López-de-Haro, L. Donati, M. Unser, A. Mu noz-Barrutia, and D. Sage, “DeepImageJ: A user-friendly plugin to run deep learning models in ImageJ,” BioRxiv, vol. 10, Jun. 2019, Art. no. 799270. 10.1038/s41592-021-01262-9 34594030

[16] A. Işın, C. Direkoğlu, and M. Şah, “Review of MRI-based brain tumor image segmentation using deep learning methods,” Proc. Comput. Sci., vol. 102, pp. 317–324, Dec. 2016.

[17] J. Long, E. Shelhamer, and T. Darrell, “Fully convolutional networks for semantic segmentation,” in Proc. IEEE Conf. Comput. Vis. Pattern Recognit. (CVPR), Jun. 2015, pp. 3431–3440. 10.1109/TPAMI.2016.2572683 27244717

[18] O. Ronneberger, P. Fischer, and T. Brox, “U-Net: Convolutional networks for biomedical image segmentation,” in Proc. 18th Int. Conf. Med. Image Comput. Comput.-Assist. Intervent. (MICCAI), in Lecture Notes in Computer Science, vol. 9351, Munich, Germany, Oct. 2015, pp. 234–241, doi: 10.1007/978-3-319-24574-4_28.

[19] K. He, X. Zhang, S. Ren, and J. Sun, “Deep residual learning for image recognition,” in Proc. IEEE Conf. Comput. Vis. Pattern Recognit. (CVPR), Jun. 2016, pp. 770–778.

[20] D. Jha, “ResUNet++: An advanced architecture for medical image segmentation,” in Proc. IEEE Int. Symp. Multimedia (ISM), Dec. 2019, pp. 225–2255.

[21] R. Gu, “CA-Net: Comprehensive attention convolutional neural networks for explainable medical image segmentation,” IEEE Trans. Med. Imag., vol. 40, no. 2, pp. 699–711, Nov. 2020. 10.1109/TMI.2020.3035253 PMC7611411 33136540

[22] J. Hu, L. Shen, and G. Sun, “Squeeze-and-excitation networks,” in Proc. IEEE/CVF Conf. Comput. Vis. Pattern Recognit., Jun. 2018, pp. 7132–7141.

[23] C. Yu, J. Wang, C. Peng, C. Gao, G. Yu, and N. Sang, “Learning a discriminative feature network for semantic segmentation,” in Proc. IEEE/CVF Conf. Comput. Vis. Pattern Recognit., Jun. 2018, pp. 1857–1866.

[24] K. Xu, M. Qin, F. Sun, Y. Wang, Y.-K. Chen, and F. Ren, “Learning in the frequency domain,” in Proc. IEEE/CVF Conf. Comput. Vis. Pattern Recognit., Mar. 2020, pp. 1740–1749.

[25] L. Marcu, P. M. French, and D. S. Elson, Fluorescence Lifetime Spectroscopy and Imaging: Principles and Applications in Biomedical Diagnostics. Boca Raton, FL, USA: CRC Press, 2014.

[26] C. Qiao, “Evaluation and development of deep neural networks for image super-resolution in optical microscopy,” Nature Methods, vol. 18, no. 2, pp. 194–202, Feb. 2021, doi: 10.1038/s41592-020-01048-5. 33479522

[27] J. C. Caicedo, “Nucleus segmentation across imaging experiments: The 2018 data science bowl,” Nature Methods, vol. 16, no. 12, pp. 1247–1253, 2019. 31636459 10.1038/s41592-019-0612-7 PMC6919559

[28] K. Sirinukunwattana, “Gland segmentation in colon histology images: The GlaS challenge contest,” Med. Image Anal., vol. 35, pp. 489–502, Jan. 2017. 27614792 10.1016/j.media.2016.08.008

[29] N. Kumar, R. Verma, S. Sharma, S. Bhargava, A. Vahadane, and A. Sethi, “A dataset and a technique for generalized nuclear segmentation for computational pathology,” IEEE Trans. Med. Imag., vol. 36, no. 7, pp. 1550–1560, Jul. 2017. 10.1109/TMI.2017.2677499 28287963

[30] N. Kumar, “A multi-organ nucleus segmentation challenge,” IEEE Trans. Med. Imag., vol. 39, no. 5, pp. 1380–1391, May 2020. 10.1109/TMI.2019.2947628 PMC10439521 31647422

[31] M. Abadi, “TensorFlow: A system for large-scale machine learning,” in Proc. 12th USENIX Symp. Operating Syst. Design Implement. (OSDI), 2016, pp. 265–283.

[32] Z. Zhou, M. M. R. Siddiquee, N. Tajbakhsh, and J. Liang, “UNet++: Redesigning skip connections to exploit multiscale features in image segmentation,” IEEE Trans. Med. Imag., vol. 39, no. 6, pp. 1856–1867, Jun. 2020, doi: 10.1109/TMI.2019.2959609. PMC7357299 31841402

[33] L.-C. Chen, Y. Zhu, G. Papandreou, F. Schroff, and H. Adam, “Encoder–decoder with atrous separable convolution for semantic image segmentation,” in Proc. Eur. Conf. Comput. Vis. (ECCV), 2018, pp. 801–818.

[34] D. Jha, M. A. Riegler, D. Johansen, P. Halvorsen, and H. D. Johansen, “DoubleU-Net: A deep convolutional neural network for medical image segmentation,” in Proc. IEEE 33rd Int. Symp. Comput.-Based Med. Syst. (CBMS), Jul. 2020, pp. 558–564.

[35] D. Jha, “Real-time polyp detection, localization and segmentation in colonoscopy using deep learning,” IEEE Access, vol. 9, pp. 40496–40510, 2021. 33747684 10.1109/ACCESS.2021.3063716 PMC7968127

[36] A. Srivastava, “MSRF-Net: A multi-scale residual fusion network for biomedical image segmentation,” 2021, arXiv:2105.07451. 10.1109/JBHI.2021.3138024 34941539

[37] J. M. J. Valanarasu, P. Oza, I. Hacihaliloglu, and V. M. Patel, “Medical transformer: Gated axial-attention for medical image segmentation,” in Proc. Int. Conf. Med. Image Comput. Comput.-Assist. Intervent. Cham, Switzerland: Springer, 2021, pp. 36–46.

[38] X. Xiao, S. Lian, Z. Luo, and S. Li, “Weighted res-UNet for high-quality retina vessel segmentation,” in Proc. 9th Int. Conf. Inf. Technol. Med. Educ. (ITME), Oct. 2018, pp. 327–331.

